# Having several options does not increase the time it takes to make a movement to an adequate end point

**DOI:** 10.1007/s00221-022-06376-w

**Published:** 2022-05-12

**Authors:** Eli Brenner, Jeroen B. J. Smeets

**Affiliations:** Department of Human Movement Sciences, Vrije Universiteit Amsterdam, Amsterdam, The Netherlands

**Keywords:** Decision making, Reflexes, Motor control, Latency, Reaction time, Choice

## Abstract

Throughout the day, people constantly make choices such as where to direct their gaze or place their foot. When making such movement choices, there are usually multiple acceptable options, although some are more advantageous than others. How much time does it take to make such choices and to what extent is the most advantageous option chosen from the available alternatives? To find out, we asked participants to collect points by tapping on any of several targets with their index finger. It did not take participants more time to direct their movements to an advantageous target when there were more options. Participants chose targets that were advantageous because they were easier to reach. Targets could be easier to reach because the finger was already moving in their direction when they appeared, or because they were larger or oriented along the movement direction so that the finger could move faster towards them without missing them. When the target’s colour indicated that it was worth more points they chose it slightly less fast, presumably because it generally takes longer to respond to colour than to respond to attributes such as size. They also chose it less often than they probably should have, presumably because the advantage of choosing it was established arbitrarily. We conclude that having many options does not increase the time it takes to move to an adequate target.

## Introduction

People make many kinds of choices in their daily life. When making some kinds of choices there is time to consider all the relevant factors, and possibly even to consult one’s friends, but other choices have to be made very quickly. When choosing whether to cross the street before an approaching car or to stop and wait for the car to pass, one may have to decide before one has had time to consider whether one would be setting a bad example for the children at the other side of the street if one were to rush across. How much time it takes to choose between the available options obviously depends on what information is considered. It may also depend on the number of options that are considered (Hyman 1953; Merkel 1885; Teichner and Krebs 1974). However, it has been noted that the dependency on the number of options is mainly an issue in situations in which the adequacy of the action is defined by the experimenter by arbitrarily associating responses with stimuli (Dassonville et al. 1999; Proctor and Schneider 2018). Moreover, the dependency disappears when an association is practiced extensively (Teichner and Krebs 1974). In natural situations, such as the example of crossing the street, neither option is inherently inadequate. The adequacy of each response depends on the position and velocity of the car, and possibly also on other circumstances (the potential costs and benefits of various outcomes; Trommershäuser et al. 2003). How long does it take to choose where to direct one’s gaze, where on an object’s surface to place one’s fingers to grasp it, or where along a forest path to place one’s foot? And how adequate are the choices that are made?

The finding that choices can take no additional time if the response is somehow compatible with the stimulus (Dassonville et al. 1999) suggests that considering multiple options may not influence the latency of responses for many of the somewhat trivial choices that constantly guide our movements in daily life. In a previous study we asked participants to tap on targets as quickly as possible. Sometimes a second target that was easier to hit appeared shortly after the original target appeared. Participants selected the second target when it saved time to do so under the prevailing circumstances. Moreover, having a choice between targets did not noticeably increase the time taken to perform the movement (Brenner and Smeets 2015a). Finding that performance did not depend on the presence of a choice suggests that the options are evaluated in parallel and selecting between them takes a negligible amount of time.

Our hypothesis is that people constantly process all viable options, and constantly update their selection of the one for which such processing provides the strongest evidence that it is suitable. They continue to process information about alternative options after the movement has started, but unless the options change after the movement has started the suitability of the selected target is only likely to increase, so the selection is not likely to change. Such constant processing allows people to make endless sequences of reasonable choices in common daily life tasks such as choosing where to place their fingers on an object that they want to pick up or where to next place their foot when walking. It is not clear whether all viable options are considered in this manner, or only ones based on characteristics that are normally relevant for the action in question rather than having been made relevant due to specific circumstances.

The present study has two aims. The first is to confirm that having a choice does not increase the latency of the response to a new option appearing. This is done by comparing the time it takes to adjust a movement to the appearance of a more attractive option, with the time it takes to adjust a movement when the original target changes position. The second aim is to provide a method to identify the circumstances in which having a choice does not increase the time taken to select an adequate endpoint. In particular, we examine what information is considered when one is forced to make fast choices: is only information that is normally relevant for guiding that kind of movement considered, or can any information be considered. We believe that knowing this is important, because despite a vast literature on decision-making, we are just starting to find out how simple everyday decisions such as where to place one’s digits when grasping an object are made (Klein et al. 2020; Schot et al. 2010), and we know almost nothing about *when* such decisions are made.

## General methods

Participants were young adults (40 male, 35 female; 65 right-handed) who volunteered to take part in this study that was conducted in accordance with approval by the Faculty’s ethical review board. Different participants took part in the four experiments: 16 in Experiment 1, 25 in Experiment 2, 10 in Experiment 3 and 24 in Experiment 4. In all experiments the participant stood in front of a large screen (115 × 88 cm; slanted backwards by 30°) onto which targets were projected from behind (800 × 600 pixels; 120 Hz; white background with luminance of 130 cd/m^2^ and CIExy chromaticity of [0.33,0.38]). Movements were tracked by attaching an Optotrak marker to the nail of the index finger of the participant’s dominant hand. This marker’s position was measured at 500 Hz.

Each session started with the participant placing his or her finger sequentially on 4 small dots on the screen to calibrate the relationship between the measured marker positions and positions on the screen, automatically accounting for the inevitable offset between the part of the finger that the participant considered to represent the position of the finger, and the position of the marker on the nail which is what we actually measured. In this paper, we will refer to the corrected value as the position of the finger. To synchronize the kinematics of the finger position with the appearance of the targets, a second marker was attached to the side of the screen. This marker’s power was switched off for some time whenever light fell on a sensor that was placed in the path of the light intended for the top left corner of the image. We programmed that corner to be dark except when new targets appeared, so the marker was registered as ‘missing’ whenever a target appeared. This was used to synchronize the moment that the target appeared with the measured finger positions to within 2 ms.

**Fig. 1:**
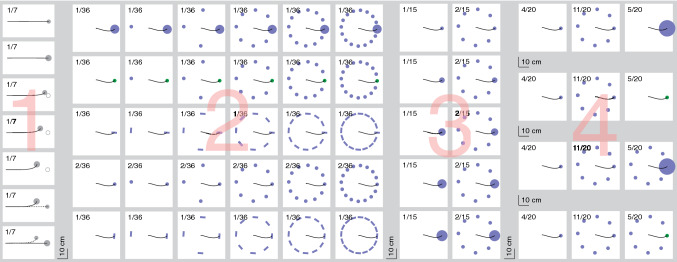
Summary of the conditions in all four experiments (experiment number indicated in pink). In Experiments 1–3 all conditions were randomly interleaved with the relative frequencies indicated in the top left corners of the graphical representations. In Experiment 4, the conditions within each of the four sessions (rows) were randomly interleaved with the indicated relative frequencies. All representations are drawn to scale. They are all drawn with the special target on the right (if present) and a schematic movement path to the right (black curve). The displays could actually be presented in any orientation with respect to the start of the movement path (the left end of the schematic movement paths in these images) and participants could move to any target. The open circles in Experiment 1 represent targets that disappeared after 200 ms (replacement conditions). Other details are explained in the specific methods sections

After the calibration, the participants’ task was to tap on as many targets as possible within 90 s (for an impression of how this was done in Experiment 1 see https://youtu.be/znqTO26MO6I). To tap on targets, participants had to lift their finger off the screen (rather than sliding their finger to the target). A tap was detected if the finger was close to the screen and decelerated intensely in the direction orthogonal to the screen (thresholds of 5 mm and 0.5 m/s^2^, respectively). After each tap there was a delay of between 22 and 60 ms before one or more (up to 16) new targets appeared. The delay varied considerably because the moment of the tap is not synchronized with when the rendering of the next image (with the new targets) starts or when the new image is actually presented on the screen. The actual moments at which new targets appeared were considered to be the beginning of the trial. By that time, the finger had sometimes already moved slightly from the position of the tap. The trials were randomly chosen from several conditions within each experiment while considering the desired relative frequencies. The conditions differed in the number of targets, what they looked like, and when and where they appeared. A summary of the conditions and their relative frequencies within each experiment is provided in Fig. 1.

Targets were presented at a given distance from the position of the finger: the target positions on a given trial were defined with respect to the position of the tap in the previous trial. Target directions were randomized, but if the randomly selected direction would place any of the targets further than 40 cm from the screen centre a new random direction was selected. If it was impossible to present all the planned targets with respect to the finger position at the moment of the previous tap, irrespective of the direction, a single target was presented at the screen centre instead. This was sometimes the case when targets were distributed evenly along a circle. Movements to this central position were not included in the analysis, but the targets for the next trial were presented relative to the finger’s position when tapping this target so they were centred on the screen.

If the position of the finger was on a target when the tap was detected the participant was considered to have hit that target. In that case a sound indicated that a target had been hit and that the participant was therefore rewarded with one (or several) points. The participants’ task was to obtain as many points as possible during each session. To motivate participants, the experiments were presented as a competition with a high-score list in which they could see their performance. Before testing, participants were allowed to practice briefly until they were sure they understood the task.

### Analysis

The participants were trying to maximize the total number of points obtained within a session, but we are more interested in how performance differs between the conditions. To quantify performance for each condition separately, we defined a measure that takes both speed and accuracy into account: the time taken per hit. To obtain this measure we determined the median time that passed from when targets of a given condition appeared until the corresponding tap was detected, and divided this time by the fraction of targets that was hit in that condition. We used the median time to not have to worry about occasional exceptionally large values that occur, for instance, if the participant does not tap hard enough so that the tap is not detected and the participant has to tap again, or if a single target appears at a position that is hidden below the participant’s arm so that it is not immediately detected. In some conditions there was a choice between different kinds of targets. For such conditions we needed to know whether the time taken per hit depended on the choice that was made, so we also split the trials on the basis of the kind of target that the participant chose. Since participants sometimes missed targets we had to infer which target they intended to hit whenever the tap was not within a target. We did so by assigning the miss to the nearest target. This allowed us to determine the time taken per hit separately for each kind of target in conditions in which the targets were not all identical. Whenever there was more than one kind of target present at the same time, we also determined how often each kind of target was chosen. Apart from these measures, we determined various measures that help us identify factors that influence the choice and how such factors may differ across individuals. All analyses were conducted for each participant separately. The figures generally show the mean (and standard error) of the participants’ individual values.

## Experiment 1

People readily adjust ongoing movements when the target of the movement changes position (Paulignan et al. 1991; Woodworth 1899). They do so with a latency of about 110 ms (Brenner and Smeets 1997). Apparently, this is fast enough to achieve the high precision of human movements (Brenner and Smeets 2018; Carlton 1981). There is no need to be aware of having to make such adjustments (Goodale et al. 1986; Prablanc and Martin 1992) and one cannot completely supress them (Pisella et al. 2000) even when they are counterproductive (Aivar et al. 2008), although counterproductive adjustments can be reconsidered slightly later (Voudouris et al. 2013). This suggests that the fastest responses do not involve considering alternatives. However, having a choice does not always delay performance (Dassonville et al. 1999; Brenner and Smeets 2015a), so there might be circumstances in which alternatives are considered without this taking additional time.

**Fig. 2:**
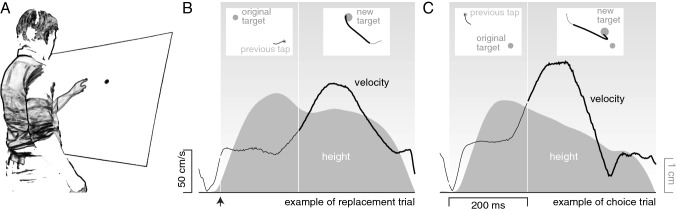
Schematic representation of the setup (**A**) and examples of trials of the two main conditions of Experiment 1 (**B** and **C**). **A**. Participants stood in front of a slanted screen and tapped on as many targets as possible within 90 s. The target for the next trial appeared soon after each tap. Sometimes, this original target was replaced (**B**) or a second target appeared (**C**) 200 ms later. **B**. The tangential velocity (curve) and height (shaded area) of the finger between two taps. This interval is divided into three sections. During the first section of this trial (white background) the finger started moving away from the screen (increasing its height) although the target had not yet appeared (the moment that the target appeared is indicated by the arrow). During the second section the target is visible and the participant’s finger starts moving towards it (the inset shows a projection of the finger’s path on the screen as a thin line). The third section starts 200 ms after the original target appeared. The original target is replaced by a target that is larger and nearer. This section lasts until the tap (thick path in inset). **C**. In choice trials the original target does not disappear when the larger and nearer target appears, so the participant could tap on either target. On this trial the finger moved to the larger and nearer target despite having to turn back to do so

To more directly determine to what extent having a choice influences the latency of responses, we compared the time that it takes to divert one’s action to an additional option that suddenly appears, with the time that it takes to divert one’s action to a similar target that replaces the existing target (Fig. 2). In the former case participants could continue their movement to the original target, or they could divert their movement to the new target that appeared later. To make it attractive to divert to the target that appeared later, that target was larger than the original one and it appeared closer to the initial finger position (so that one can expect to be able to tap it faster without making more errors). The difference between the latency with which the finger is diverted towards such targets, and the latency with which the finger is diverted towards a new target that replaces the original one, might provide us with information about how much time it takes to select between all considered options, following the reasoning originally proposed by Donders (1868; reprinted in English as Donders 1969).

### Specific methods and analyses

There were seven conditions (see left column of Fig. 1). The two main conditions (*choice* and *replacement*; sixth and fifth panels in Fig. 1) have already been introduced by the examples in Fig. 2. In the main *choice* condition (Fig. 2C), the original target remained present when a new target appeared, so that the participant had to choose between the two. The new target was nearer and larger and was therefore easier to hit. In the main *replacement* condition (Fig. 2B), the same new target replaced the original target, so there was no choice to make. This main *replacement* condition differed from classical target jump paradigms in that the new target was larger than the original target and the change in position was quite large. To examine whether these differences influence the response latency, we included two additional *replacement* conditions with more modest changes in size and position. Moreover, to make sure that participants actually made a sensible choice rather than just switching to a new target whenever one appeared, we added a second *choice* condition in which the new target was more difficult to hit rather than easier to hit. In the two final (*no change*) conditions, no new targets appeared and participants simply had to hit the original target. There were two possible targets that were identical to the original targets of the two choice conditions.

The targets were black disks that could have various diameters and were presented at various distances from the previous tap. There were two possible original targets: ones that were easier to hit (5.3 cm diameter at a distance of 26.4 cm) and ones that were more difficult to hit (3.5 cm diameter at a distance of 35.2 cm). In the conditions in which the target was replaced or a second option appeared, this always happened 200 ms after the original target appeared. We chose this time to ensure that the movement will have started by the time a response would be visible, while still leaving enough time to respond. To make it easy for us to isolate adjustments to the movement, the new target always appeared slightly off an imaginary line connecting the initial finger position to the original target’s position. For each condition, the magnitude of the angle by which the new target was off the line to the original target was fixed, but it could be either in the clockwise or the counterclockwise direction. In the three *replacement* conditions, the original target was always difficult to hit. It was replaced after 200 ms by one that was easier to hit: a 4.1, 4.7 or 5.3 cm diameter target that was at a distance of 32.3, 29.3 or 26.4 cm and was off the line to the original target by 6, 8 or 10°, respectively. In the two *choice* conditions the new target appeared 10° off the line to the original target. In the main *choice* condition, the original target was difficult to hit and the new target was easy to hit. The position and size of the new target precisely matched that of the new target in the main *replacement* condition, so that the only difference was that the original target did not disappear. In the other *choice* condition the order in which the targets appeared was reversed, so the original target was easy to hit.

The participants’ task was to hit as many targets as possible within each session. After some practice, participants took part in 3 sessions, with a short break between the sessions. The condition was selected at random after each tap (all 7 conditions had equally probability). Apart from determining the time taken per hit for each condition and kind of target chosen, we also determined the mean position of the finger at the moment that the second target appeared for the main *choice* condition. We did so separately for trials in which participants selected the original (difficult) target and the new (easy) target, to see whether the finger’s position when the new target appeared influenced participants’ choices. The finger’s position was determined with respect to its position at the time of the previous tap (which is the position that determines where the targets appear). For our analysis, we used a coordinate system with one axis in the direction of the original target and the second axis in the orthogonal direction, whereby the side on which the new target appeared was positive.

The finger’s velocity in the direction orthogonal to that of the original target was used to evaluate how much longer it took to respond to the new target when doing so involved making a choice than when one was simply responding to a target jump. This velocity was determined for each trial of the main *choice* condition and the three *replacement* conditions. Since whether new targets appeared in a clockwise or counterclockwise direction was determined completely at random, there could be different numbers of new targets in the two directions, so we averaged the orthogonal velocities separately for each direction before averaging them across the two directions. Doing so ensures that any systematic bias to deviate in the clockwise or counterclockwise direction, irrespective of the new target, could not influence our interpretation.

The finger often started moving away from the screen before the targets appeared (see example in Fig. 2B). To ensure that selecting targets in which the finger is moving in a certain manner before the new target appears does not influence our estimate of the response to the new target (in the choice condition), we included all trials in this analysis, irrespective of which target was chosen. Since including trials in which participants did not divert their movement to the new target obviously reduces the average magnitude of the response, we then divided the finger’s average velocity orthogonal to the direction to the original target by the fraction of trials in which the new target was chosen. This procedure, and the kind of artefacts that occur when only trials that end at the new target are considered, are illustrated in Appendix 1. We refer to the average finger velocity orthogonal to the direction to the original target, corrected for the fraction of trials in which the original target was chosen, as the *response.*

## Results

**Fig. 3:**
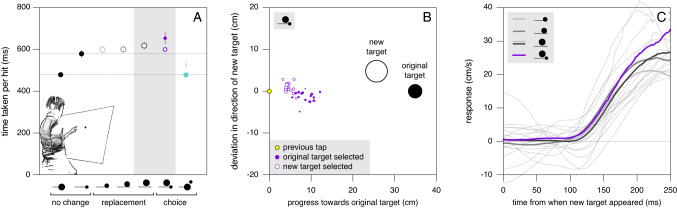
Results of Experiment 1. **A**. Time taken per hit for (from left to right) the easy and difficult target to hit (no change), three increasingly easier targets that replaced a difficult target (replacement), and choices between the easy and difficult target when the difficult or easy one appeared first (choice). For the two choice conditions, times are presented separately for tapping the original (solid symbols) and the new (open symbols) target. Symbol area indicates the number of trials involved. Error bars are standard errors across 16 participants. Dotted lines indicate the values for the two conditions with no change. The grey background indicates the two main conditions. **B**. Average positions of individual participants’ fingers at the moment that the second target appeared in the main choice condition. **C**. Responses to the three target replacements (contrast of curves increasing from light grey to black with the magnitude of the change) and to being given the opportunity to choose an easier target (purple curve). Thin grey curves show the 16 individual participants’ average responses for the main replacement condition

As expected, the time taken per hit in the *no change* conditions was shorter for large targets nearby than for small ones further away (solid black disks in Fig. 3A). In the other five conditions, the time taken per hit mainly depended on the original target. Although the new target was larger and closer than the original target in the three *replacement* conditions (grey and black circles) and in the main *choice* condition (purple circle), the time taken to hit this target was not shorter than it would have been if there were no change (upper dotted line). On average, the time taken per hit was quite similar in the main *choice* condition (average of purple symbols) and in the main *replacement* condition (black circle). In the main *choice* condition, the new easy target was chosen on slightly more than half the trials. When the new easy target was chosen the time taken per hit was shorter than when it was not (purple circle below purple disk). When it was not chosen the time per hit was substantially larger than for the corresponding *no change* condition (compare purple disk with upper dotted line), despite the fact that participants were more likely to choose the original target if they were closer to it at the moment that the new target appeared (Fig. 3B). Simply having a new target appear did not automatically increase the time taken, because when a target that was more difficult to hit was added (such targets were seldom chosen) participants did not take longer to hit the original target than when no new target was added (compare solid turquoise symbol with lower dotted line).

The main purpose of the first experiment was to determine how much longer it took participants to respond to a new easy target being presented alongside the original difficult target (in the main *choice* condition) than to a new easy target replacing the original difficult target (in the main *replacement* condition). This difference could be considered to be a measure of how long it takes to select between the two options once they have both been processed, assuming that they are processed simultaneously. Individual participants’ responses in the main *replacement* condition are shown by the thin curves in Fig. 3C. The mean of the 16 participants’ responses is shown by the thick black curve. In the main *choice* condition participants chose the new target on about half the trials. Individual responses in the main *choice* condition were corrected for the fraction of trials in which the new target was chosen, and averaged to give the mean response shown by the purple curve. The two mean responses are very similar, both in terms of latency and in terms of vigour. The response was certainly not later in the main *choice* condition, so it clearly did not take a substantial amount of additional time to select between the two options. In both cases the latency was just above 100 ms, which is similar to what is found for small, inconspicuous displacements (Brenner and Smeets 2015b). It is therefore not surprising that replacing the original target by more similar targets resulted in responses with similar latencies (grey curves). The responses to replacing the original target by more similar targets are less vigorous (from about 200 ms after replacing the target), in accordance with the required correction being smaller because the position changes less.

## Discussion

In the two choice conditions, participants really did choose rather than simply responding to any new target that appeared: they considered how easy it was to hit the targets (very seldom selecting the new target if it was more difficult to hit; small turquoise symbol in Fig. 3A) and considered the position of their finger when making their choice (Fig. 3B). The latter finding is in line with the results of our previous study (Brenner and Smeets 2015a). To determine how much time it takes to choose between the two alternatives, we compared the two conditions in which the same new target appeared: the main *choice* condition and the main *replacement* condition. The latency of the responses was similar in both conditions (Fig. 3C), as was the average time taken per hit (Fig. 3A). Thus, having a choice does not appear to delay the response at all.

Comparing the *choice* conditions with the *no change* conditions that have the same original target shows that although not choosing the new target when it was more difficult to hit did not delay the finger (turquoise disk on lower dotted line in Fig. 3A), not choosing the new target when it was easier to hit did (purple disk above upper dotted line). The latter finding on its own does not prove that having a choice delays the response, because the new target could specifically not have been chosen when the movements were slower from the start. However, the finger had actually moved *further* towards the original target by the time the new target appeared on trials in which the new target was not chosen (Fig. 3B). This might be because the adjustment towards the new target can only start about half way through the movement (100 ms after the new target appears, so 300 ms after the original target appears, with the time taken per hit being about 600 ms). If the finger has travelled too far within this time it will be disadvantageous to choose the new target because the adjusted path will become very long (see Fig. 2C).

So why did it take longer to hit the original difficult targets in the main *choice* condition than to hit identical targets in the *no change* condition (purple disk above upper dotted line in Fig. 3A) despite the fact that the finger had moved particularly far when the choice was presented (Fig. 3B) and the choice itself did not take any additional time (Fig. 3C)? A possible explanation is that when a new item appears near the hand’s path it is treated as an obstacle, and therefore slows down the movement in the manner that having an object near the hand’s path does when it is there from the start (Biegstraaten et al. 2003; Mon-Williams et al. 2001; Tresilian 1998) even if it is a virtual object (Carr et al. 2008). Movements are quickly adjusted when an obstacle moves (Aivar et al. 2008) and the gain of reflexive responses to mechanical perturbations is influenced by the presence of obstacles (Nashed et al. 2012, 2014), so it is not inconceivable that the speed with which one moves would decline if a potential obstacle suddenly appears.

## Experiment 2

The results of Experiment 1 suggest that people process information about the two options at the same time, and that it takes no additional time to choose between the two targets. When doing so, people consider the position of their finger, as well as the targets’ sizes and distances. Does this extend to having more options, as one often has in daily life? How about choices between targets that are further apart and ones based on other measures than distance and size? Are such fast choices limited to spatial features that normally guide human movements?

In the second experiment, we examined how efficiently people can choose between different numbers of options. Targets were distributed evenly along a circle centred on the position of the previous tap. The targets all appeared simultaneously. One of the targets could be different than the rest in one of three attributes. Do participants select this special target when it is likely to be advantageous to do so? This study differs fundamentally from most previous studies involving choices between many targets (see Proctor and Schneider 2018 for a recent review of such studies based on reaction times) in that the correct choice is not something arbitrarily defined by an instruction. As in many situations in daily life that require fast decisions between several options, many of the options are acceptable. Do people make fast and reasonable choices under such circumstances?

The three target attributes that we examined are size, orientation and colour. Since we have seen that it takes people almost no additional time to reach a suitable target when they could select between two adjacent targets, we anticipated that they would also be able to do so when there are more than two targets. To examine whether they could also do so on the basis of other spatial attributes than size and position, we examined whether participants would quickly choose an elongated target that is oriented more favourably than the others. To examine whether they could also do so on the basis of an attribute that is not normally relevant for guiding human movements, we examined whether participants would quickly choose a target of a colour that is arbitrarily associated with a higher ‘value’.

### Specific methods and analyses

**Fig. 4:**
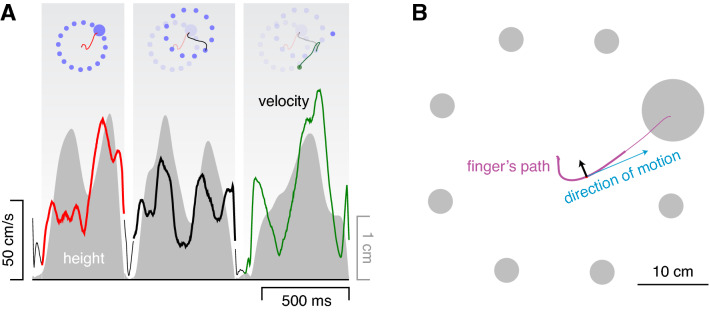
Methods of Experiment 2. **A**. Three consecutive trials (from left to right) with first 16 targets of which one was larger (size set), then 12 standard targets (control set), and finally 2 targets of which one was green (colour set). As in Fig. 2, the curves and left axis indicate the finger’s tangential velocity and the shaded area and right axis indicate the finger’s distance from the screen. The background is white and the curves are thin and black for the time between the previous tap and the appearance of the new set of targets. The schematic representation at the top of each part shows the target configuration and the finger’s path. Previous target configurations are faintly visible to clarify the spatial relationships but were not visible during the experiment. **B**. We determined how the finger’s path curved in the direction of the special target by calculating the acceleration orthogonal to the instantaneous direction of motion (black arrow). This was done at each instant, only considering the component of motion parallel to the screen. Acceleration in the correct direction for reaching the special target was considered positive, irrespective of which target was chosen

Each of the 25 participants took part in four sessions, with short breaks between the sessions. There were thirty conditions that were randomly interleaved. These thirty conditions consisted of five sets (defined by the kinds of targets that were present), each including conditions with 1, 2, 4, 8, 12 or 16 targets. The targets were equally spaced along a 17.6 cm radius circle, centred with a random orientation around the position of the latest tap (see example in Fig. 4A). In three sets of conditions the targets were disks and in the other two they were bars. In two sets of conditions with disks and one with bars there was one special target (upper three rows in Fig. 1). In the other two conditions all targets were the same (lower two rows in Fig. 1). Unless mentioned otherwise, the targets were blue (27 cd/ m^2^ with a CIExy chromaticity of [0.29, 0.29]). They were either 3.5 cm diameter disks or 5.3 by 1.8 cm bars that were oriented tangentially with respect to the circle. In the *size* set the special target was a disk with a diameter of 8.8 cm rather than 3.5 cm. In the *colour* set, the special target was a green (70 cd/m^2^ with a 1931 CIExy chromaticity of [0.34, 0.45]) rather than a blue disk. In the *orientation* set, the special target was a 5.3 by 1.8 cm bar that was rotated by 90° so that it was oriented along the movement path rather than orthogonal to the path.

Participants were told in advance that green targets were worth double points. The green targets were identical to their blue counterparts except for their colour, so there was no difference in terms of how easily one could hit them, but hitting green targets was advantageous in terms of achieving a high score even if it took more time to reach them. Given that the variability of movement endpoints is usually oriented in the movement direction (e.g. van Beers et al. 2004), we expected the bar to be easier to hit when it was oriented along the movement path. Thus, in all cases in which there was a special target it was advantageous to choose that target, but for different reasons. The condition on each trial was chosen at random from all 30 possibilities, with the control condition with disks being twice as likely as the other four conditions because it provides the baseline for both the conditions with a large disk and the ones with a green disk.

Presumably, the position of the hand influences the choice of target (Fig. 3B) because the magnitude of the required adjustment is considered. We therefore expected targets close to an advantageous target to be chosen particularly infrequently, because a smaller adjustment is needed to divert the movement towards the advantageous target when one is moving in the direction of a nearby target. To examine whether this is the case, we determined the probability of a target being chosen as a function of its distance from the special target (for trials in which there was a choice between two kinds of targets). To account for there being different numbers of targets on different trials, and to not have to differentiate between distances in the clockwise and counterclockwise direction, we expressed the probability of a target being chosen with respect to chance.

To examine whether the variability in how likely participants were to choose the special target is related to differences in the times they took for the various targets, we determined the correlation across individuals between (1) the difference in time per hit when there was only a single special target and when there were multiple standard targets without a special target, and (2) the fraction of trials in which they chose the special target when there was a choice. This was done separately for special targets that differed in size, orientation or colour (after averaging the values for the different numbers of targets for each participant).

Differences in reaction time are often used to determine how quickly participants can choose between multiple options. In our experiment, participants lifted their finger before the targets appeared, so we could not rely on motion onset to determine when a choice was made. To determine when the choice was made we examined when the finger’s path started curving towards the special target. To determine the curvature towards the special target, we first determined the direction in which the finger was moving and then determined the finger’s acceleration orthogonal to that direction (see Fig. 4B). We could not determine when it started curving towards the chosen target, because the choice itself depends on how the finger is moving at the time (Fig. 3B), so the average trajectory would curve towards the chosen target from well before participants could have responded to the targets appearing (see Appendix 2). For the same reason, we had to include all trials, rather than only ones that ended on the special target. As long as participants choose the special target more often than other targets, curving towards other targets largely averages out, which reduces the magnitude of the apparent response but leaves the timing intact.

We determined the finger’s acceleration orthogonal to the direction in which it was moving for each moment during the movement (2 ms intervals). The finger’s velocity and acceleration at each moment were determined by fitting a second order polynomial to the measured positions between 25 ms before and 25 ms after that moment (Savitzky–Golay filter with a window size of 50 ms). Acceleration in the direction of the special target (with respect to the instantaneous motion) was considered positive. In the example in Fig. 4B the curvature is positive because the acceleration is towards the same side of the blue arrow as the special target. We used the average acceleration at each moment from when the target appeared to determine how long it took to adjust movements towards each kind of target when there was no choice.

When there were multiple targets, simply averaging the acceleration across trials is not enough to ensure that all curvature that is not an adjustment towards the special target averages out, because the acceleration is not necessarily equally likely to be positive as negative when the movement is not directed towards the special target. For example, in Fig. 4B five of the eight targets are in the same direction as the acceleration, so even if the special target were at a different position the acceleration would be more likely to be positive than negative. To compensate for this, we defined the *correction* at each moment as the acceleration (*a*) scaled by the fraction (*f*) of targets that were at the opposite side of the line representing the direction of motion (indicated by the blue arrow in Fig. 4B) than the direction towards which the acceleration was directed (*correction* = 2 *a f*). This meant that if the acceleration was towards the side on which all targets were situated the curvature was ignored. The necessity of including all these steps in the analysis is illustrated in Appendix 2. The mean correction at each moment was determined for each participant separately, and the mean and standard error across participants is shown for the first 300 ms after new sets of targets appear.

The latency of the correction was determined using the extrapolation method on averaged data (Brenner and Smeets 2019): the points at which the average response reached 25% and 75% of its peak value were determined, and the time at which a line through these two points crossed a response of zero is considered to be the latency. To obtain an estimate of the confidence in the latencies we used a bootstrap method whereby the mean curvature for individual participants was based on randomly selecting trials from the available data (the same number of trials as were available for that participant, but sampled at random) and averaging the curvature on such trials first within and then across participants. The latency was determined from these average curvatures. We did this 1000 times, and plot the median latency as well as the ranges containing 50% and 95% of the bootstrapped latencies.

## Results

**Fig. 5:**
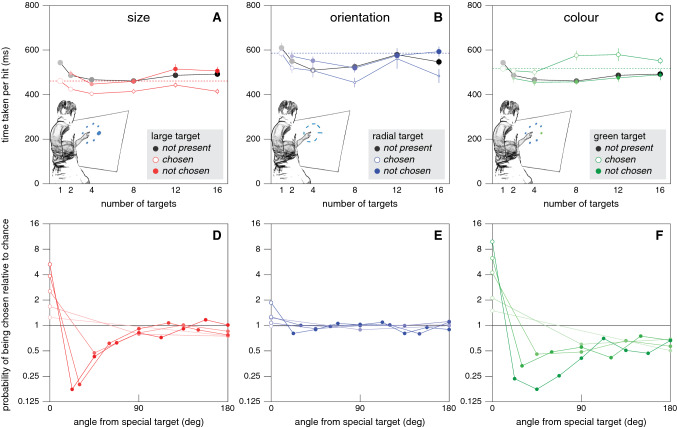
Results of Experiment 2. Symbol colour indicates the set of conditions: red for size, blue for orientation, green for colour, and grey for the corresponding control sets. **A-C**. Median time taken per hit for sets with each kind of special target and the corresponding control set. For all conditions with a special target the time is determined separately for attempts to tap the standard (solid symbols) and special (open symbols) target. Error bars are standard errors across participants. Symbol area indicates the fraction of trials involved. The dashed lines indicate the time taken when there is only a special target, to make it easier to evaluate the choices. The grey symbols in A and C represent the same data. **D-F**. The probability of tapping on a target as a function of the target’s position with respect to the special target. Lines connect points for the same condition. Note that the probability scale is logarithmic

In accordance with selection between the options taking no additional time, in the control conditions the time taken per hit did not simply increase with the number of standard targets (grey symbols in Fig. 5A–C). On the contrary, having more than one equivalent option was clearly advantageous, although increasing the number of options beyond 8 does not appear to be advantageous. Presumably, having several options allows participants to choose the one that is easiest to reach at that moment, and the advantage that this offers far outweighs the cost of making the choice, if there is any cost. How about choices between different kinds of targets?

For the size set, the results are clear. When the chosen target was large it took less time to hit it (open symbols below solid symbols in Fig. 5A). When a standard target was chosen it did not matter whether there was a larger target present (no systematic difference between solid red and grey symbols). The probability of the participant choosing the large target was well above chance (values larger than one for angle zero in Fig. 5D), mainly at the expense of choosing nearby targets (that were therefore chosen with a probability below chance). The probability of choosing the targets that were more than 90° from the special target was close to chance (so they were as likely to be chosen as in the absence of a special target). This influence of the separation seems to be independent of the number of targets.

For the orientation set, the tendency to choose the special target was much weaker, but the interpretation is similar. As anticipated, it took less time to hit radially oriented targets than to hit tangentially oriented ones (open symbols lower than solid ones in Fig. 5B), but the difference was smaller than for the size set. Importantly, the benefit of the radial target orientation was usually smaller than that of having more targets (most filled symbols are below the dashed line), so it is unlikely to often be beneficial to specifically select the radially oriented target. Consequently, participants hit these special targets only slightly more often than one would anticipate by chance (Fig. 5E). Again, when they did not choose the special target the time taken per hit did not depend systematically on the presence of a special target.

For colour, we have to interpret the time taken per hit differently, because the benefit of selecting the green target is not in the time taken per hit, but in the fact that hitting the green target is worth double points. The grey points for the control condition in Fig. 5C are the same data that are shown in Fig. 3A. One rather unexpected finding is that participants hit single green targets faster than they did single blue targets in the corresponding control condition (a difference of 27 ms; a post-hoc paired t-test suggests that this might be a real effect: *t*_24_ = 2.31, *p* = 0.03). Participants were faster for green targets despite the luminance contrast being higher for blue targets (and colour not having to be determined to guide the movement when there is only one target). The time taken per hit was lower and the fraction of targets hit was higher, so this is not just a better choice with regards to the speed-accuracy trade-off. We will confirm and discuss this finding in Experiment 4.

When there was a green target but it was not chosen, the time taken to hit the target was similar to that in the corresponding control condition. When the green target was chosen from several options it took slightly longer to hit it (open green symbols above solid ones). That the time was not shorter as it was when the special target differed in size or orientation is not surprising, because it was not easier to hit the green targets. But why was the time longer? Green targets were selected on more than half the trials (open symbols larger than filled ones) despite it taking longer to reach them, because it was worth taking more time to obtain double points. Consequently, not only targets near the green target were chosen less frequently than one would expect by chance, but even targets in the opposite direction (Fig. 5F). It would presumably have been advantageous to choose the green target even more often, because the benefit in terms of points is much larger (a factor two) than the cost in time taken. One reason for not doing so could be that the additional time that it takes to make a choice is not negligible in this case, maybe because the colour is irrelevant for the movement itself as we argued in the introduction.

**Fig. 6:**
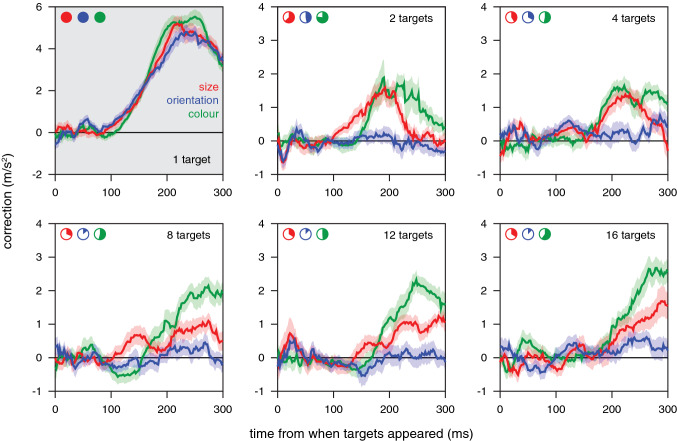
Response of the finger’s path in the direction of the special target in Experiment 2. Mean and standard error of the 25 individual participants’ mean values for each set of conditions (curves) and each number of targets (panels). The filled parts of the pie charts at the top left of each panel show the fraction of trials on which the special target was chosen in the condition indicated by the pie chart’s colour

**Fig. 7:**
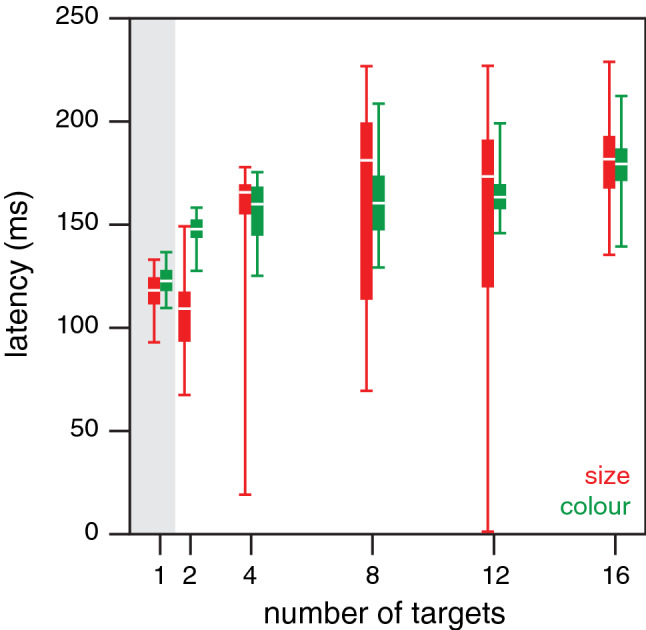
The latency of the corrections to the finger’s path (from when the targets appear) in Experiment 2. Values based on bootstrapping the data underlying Fig. 6. Horizontal white lines show the median latency, bars show the inter-quartile range, and vertical lines show the range including 95% of the 1000 samples

To determine how long it took our participants to make a choice we examined their fingers’ paths to estimate when such paths curved towards the special targets. It took about 100 ms for the finger to start moving towards the target when there was a single target, irrespective of the target’s size, shape or colour (curves in top left panel of Fig. 6). When there were several targets, so that a choice had to be made, the precise latency of the response was less clear (curves in other panels of Fig. 6). The responses for colour (green curves) were not very different from those for size (red curves). The advantageously oriented targets were not chosen frequently enough for this method to reveal a clear response for orientation (blue curves). For the size and colour sets, the estimated latencies of the corrections (as estimated in the manner described at the end of the "*Specific methods and analyses"*) were very variable (Fig. 7), but the median latencies were quite similar. The latency was about 50 ms longer when there were many targets than when there was only one target (about 170 and 120 ms, respectively). The most important finding is that it did not generally take much longer to make a choice based on colour than one based on size, as one might expect if the relationship between the feature and the adequate response were critical (here comparing evaluating the arbitrary relationship between colour and points with comparing the usual relationship between size and how fast one can move).

To evaluate whether participants who would benefit more from choosing the special target did so more often, we examined whether the tendency to choose the special target depended on the advantage of choosing the special target. This advantage was quantified as the difference in time per hit between the condition with only the special target (dashed lines) and the conditions with only standard targets (grey symbols in Fig. 5A–C), averaged across the different numbers of targets (excluding the condition with only one standard target). This value could be negative, indicating a disadvantage. The correlation between the advantage of choosing the special target and the fraction of trials in which participants chose the special target when there was a choice was 0.36 (*t*_23_ = 1.86, *p* = 0.076) for size, 0.31 (*t*_23_ = 1.58, *p* = 0.13) for colour, and 0.26 (*t*_23_ = 1.30, *p* = 0. 21) for orientation. Thus, they were all positive, as one might expect, but none significantly so.

## Discussion

That having several options is advantageous is presumably partly because certain movements are easier than others, and having the choice allows participants to choose an easy one. Moreover, it is easier to make small adjustments than large ones, so it is easier to adjust movements when there are many options simply because there is more likely to be a target near the direction in which one is moving. There appears to be a tendency for the time taken to hit the targets to increase slightly when more than 8 targets are presented, possibly because the presence of additional objects near the target interferes with movement execution (Tipper et al. 1997). It also took longer to tap on the green target when there were many blue targets present than when it was presented alone (open symbols above dashed line for large numbers of targets in Fig. 5C), which is consistent with having other targets nearby slowing the movements down. However, we also found that it takes more time to *adjust* the movement when the green target is not presented alone (Fig. 7).

Overall, participants appear to be making reasonable choices. In the conditions of the size set, they choose the larger target at the expense of neighbouring targets, reducing the mean time per hit by doing so. The hand was already moving when the targets appeared, so it was presumably only worth adjusting the movement towards the larger target if the original direction was within 90 deg of that target (Brenner and Smeets 2015a; Hudson et al. 2007). In the conditions of the orientation set, participants seldom switched to the radially oriented target despite being able to reach such targets faster, presumably because it was not worth the cost of adjusting the movement even by a small angle (the benefit of a radial target orientation is clearly smaller than that of having multiple targets to choose from; Fig. 5B).

In the conditions of the colour set, the rather small increase in the time taken to hit the special, green targets (Fig. 5C) and the short latency with which the movement path was adjusted towards these special targets (green bars in Fig. 7) suggest that it may have been advantageous to adjust the movements to hit the special targets much more often. The latency with which movements are adjusted appears to be quite similar when the special target was green as when it was large, although it might be larger for colour than for size when there are two targets (Fig. 7). The latencies for adjusting movements when faced with such a choice are not known very precisely, so we checked this by performing a supplementary experiment (described in Appendix 3) to compare the responses to the appearance of new targets (as in the main *choice* condition of Experiment 1) that were either larger and therefore easier to hit or had a different colour (this time red) that was arbitrarily assigned more points. In this supplementary experiment, the task was to move a cursor to targets on a computer screen. The results confirm that it takes slightly longer to respond to colour than to size (Veerman et al. 2008). Since it takes less than 50 ms longer, participants could probably have obtained more points by choosing the green target more often in Experiment 2. Thus, choices based on colour were fast, but opportunities to obtain more points may have been missed.

The extent to which participants adjusted their movements when the targets appeared might not only depend on the direction in which the hand was moving (accounting for the pattern in Fig. 5D) but also on how fast they were moving (see horizontal separation in Fig. 3B). It might therefore have been worth slowing down a bit to have more time to adjust the movements when there were green targets (Hudson et al. 2007). Since all the conditions were randomly interleaved only one in six trials had a green target, so delaying movements to choose more green targets may not be optimal because it would also make it take longer to hit other targets. To perform optimally in this respect, participants would have to consider the frequency of occurrence of green targets, which influences how much time it is worth losing by tapping less quickly to select more green targets. In Experiment 4 we will examine whether there is evidence for any influences of the set of trials within a session on the speed at which movements in the same conditions are made. However, before doing so we checked that participants considered the target’s size, rather than just selecting the largest target.

## Experiment 3

The results of Experiment 2 support the idea that options can be considered in parallel, with the choice itself taking almost no additional time. This even seems to be the case when the choice is based on an arbitrary relationship. However, the choice itself may be less adequate for the arbitrary relationship that we tested. The complicated pattern of advantages of having more or fewer targets makes it impossible to determine the optimal choice of target in Experiment 2. We feel confident that it would have been advantageous to choose the green target more often than participants did so. They may have chosen the larger (and differently oriented) targets whenever it was advantageous to do so. If so, their preference for the large target should increase with the size difference. To check whether the size is evaluated on the fly, rather than just recognizing that one target is larger, we conducted a very similar experiment to Experiment 2, but only including variations in size and only with 1 or 8 targets. The main manipulation was the size itself: we used several different sizes.

### Specific methods

The methods were identical to those of Experiment 2 except for the conditions and that each participant did 5 rather than 4 sessions. Targets could have a diameter of 3.5 cm (the standard size), 5.3 cm, 7.0 cm, 8.8 cm (same as the large target of Experiment 2) or 10.6 cm. There were ten conditions: five with a single target and five with eight targets. When there was a single target, it had one of the five possible sizes. When there were eight targets, seven had the standard size and one was special. The special target had one of the five possible sizes. When there were eight targets and the special target had a diameter of 3.5 cm, all the targets had the same diameter, so actually none of the targets was special. Again, the targets were equally spaced while the whole configuration had a random orientation (Fig. 1). The conditions were presented in random order, except that the conditions with eight targets were presented twice as frequently as the ones with a single target. The data were analysed in the same manner as those of Experiment 2. There were ten participants.

## Results and discussion

**Fig. 8:**
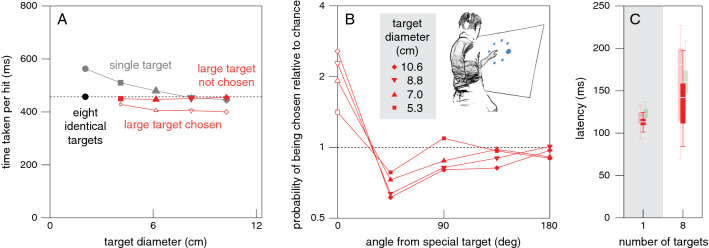
Results of Experiment 3. **A**. Median time taken per hit for each combination of targets. Grey symbols: single targets of various sizes (indicated by different symbols). Black symbol and dotted line: eight standard targets. Red symbols: seven standard targets with one larger target. Open red symbols: trials in which participants tapped on the larger target. Solid red symbols: trials in which they tapped on a standard target. Symbol area indicates the fraction of trials involved. **B**. The probability of tapping on a target as a function of its position with respect to the larger target. Symbols indicate the size of the larger target as indicated by the horizontal position in A. Lines connect points for the same target size. **C**. Latency of corrections to the finger’s path, averaged over all target diameters, in the same format as in Fig. 7. The estimated latencies for one and eight targets in Experiment 2 are faintly visible in the background for comparison

As in Experiment 2, it took less time to hit one of eight standard targets (black disk in Fig. 8A) than to hit a single standard target (grey disk). Also, as expected, the time taken to hit single targets decreased with target size (grey symbols). For the four conditions in which there was one larger target and seven standard ones (red symbols), the time taken to hit the standard targets was the same as the time taken to do so when there were eight standard targets (solid red symbols follow the dashed line), whereas the time taken to hit the larger target was shorter (open red symbols below solid red symbols). It was also shorter than the time taken when the larger target was presented on its own (open red symbols lower than the corresponding grey symbols), and it was lower the larger the target. This is all in accordance with the results of Experiment 2.

The main question in this experiment was whether the choice would also depend systematically on the size of the target. It clearly did: as target size increased the probability of tapping on the larger target increased (values at angle zero in Fig. 8B) and the probability of tapping on neighbouring targets decreased (values at 45 deg and 90 deg), in accordance with the benefit of selecting the large target. The correlation between the tendency to choose the large target and the expected benefit of doing so (averaged over the different target diameters) was 0.50 (*t*_8_ = 1.63, *p* = 0.14). Thus, as in Experiment 2, the correlation was positive but not significantly so. The latencies with which the finger’s path was adjusted to the presence of a larger target were similar to those in Experiment 2. Again, the ranges for the two numbers of targets overlap considerably. Here, the difference between the median latencies for a single and many targets is only about 30 ms (Fig. 8C).

Three conditions were present in both Experiments 2 and 3: a single 8.8 cm diameter target, eight 3.5 cm diameter targets, and seven 3.5 cm diameter targets accompanied by one 8.8 cm target. Comparing performance for the corresponding conditions in the two experiments we see that the values are very similar (see values at 8 targets in Fig. 5A and at 8.8 cm diameter in Fig. 8A, including the position of the dashed line). This suggests that performance is not too sensitive to the other conditions that are presented within the session. Thus, for instance, the fact that size is not the only factor to consider in Experiment 2, whereas it is in Experiment 3, and that the frequency of trials in which there was a choice differed between the experiments, does not appear to make much difference. Our final experiment was designed to examine to what extent the possibility of an advantageous option being present influences performance.

## Experiment 4

The results of Experiment 3 suggest that the extent of the advantage is considered rather than only which target is the most advantageous. The fact that one might need to consider certain attributes when making one’s choice could make one move differently. For instance, participants may intentionally move more slowly if they anticipate that a particularly advantageous option that they should not miss, such as a green target that is worth more points, might sometimes appear. Participants may consider the instructions and the conditions they encountered within a session (and possibly previous sessions) to quickly develop a consistent movement pattern. Alternatively, they may change their behaviour after each tap on the basis of the circumstances during that tap, leading to serial dependencies across trials (Brenner and Smeets 2011; Cheng et al. 2011; de Lussanet et al. 2001; Volcic and Domini 2018). In both cases, one might expect participants to move faster in sessions in which there is never a choice between different kinds of targets. In the latter case, one might expect to observe that participants consistently slow down after having missed a target or an advantageous option.

In Experiments 2 and 3, all conditions were interleaved and there were too many conditions to meaningfully examine serial dependence. In Experiment 4, rather than presenting many conditions within each session, we only presented three conditions within each of the four sessions. Two conditions were presented in all four sessions. We examined whether the time taken per hit in these two conditions depended on whether there was a choice between different kinds of targets in the third condition within that session, and if so whether it depended on the attribute that was the basis for the choice (size or colour). We also examined whether differences between sessions could be the result of direct serial dependence.

### Specific methods and analyses

The standard targets were the same blue 3.5 cm diameter disks as in Experiments 2 and 3. With 24 participants, we could fully counterbalance the order of the four experimental sessions, so that each possible order of the four sessions was performed by exactly one participant. In each of the four sessions, three conditions were randomly interleaved. The two conditions that were present in all four sessions are the *single standard target condition* (20% of the trials) and the *eight standard target condition* (55% of the trials). We presented the *eight standard target condition* most frequently because we anticipated that if having a choice between different kinds of target on some trials influences performance when there is only one kind of target, it is most likely to affect performance in the condition in which the participant can choose. The third condition (25% of the trials) differed between the sessions. It always contained one special target: either a *large* target (15.8 cm diameter) or a *green* target (standard size). The special target was either presented *alone* or together with 7 standard targets so that there was a *choice* between different kinds of targets. We identify the four sessions by the special target that is present in this third condition: *large alone*, *green alone*, *large choice*, and *green choice.*

To make sure that participants would often choose the special target, we made it even more attractive than in the previous experiments: the large target was larger than in any of the previous experiments and the green target was assigned ten points (i.e. equivalent to hitting ten standard targets). Before taking part in the experiment participants practised tapping on targets for 30 s with only the two conditions without a special target (*single standard target condition* and *eight standard target condition*). The data of this practice period were not analysed.

Most of the analyses were very similar to those in Experiments 2 and 3. We examined the median time taken per hit for each condition, the likelihood of selecting the special target when it was present in the two choice sessions, and the correlation across individuals between the expected advantage of choosing the large target and the fraction of trials in which it was chosen. The correlation now had to be done by comparing trials from different sessions. The expected advantage of choosing the large target (when presented together with 7 standard targets) was the difference between the time per hit when one had to hit the large target (large alone session) and the time per hit in the *eight standard target condition* (averaged across the four sessions). The tendency to choose the large target when it was present was obviously determined from the condition in which there was one large target and seven standard targets (large choice session).

The most important comparison in this experiment is that between the times per hit in the four sessions for the two conditions that were present in all sessions: the *single standard target condition* and the *eight standard target condition*. For each of these conditions we conducted a two-way repeated measures analysis of variance to determine whether the time per hit depended on the targets that were present in the third condition. The third condition differed between the four sessions in two ways, resulting in two factors for the analysis of variance: whether the special target was presented alone or together with seven standard targets (*alone* or *choice*) and the kind of special target (*large* or *green*). In addition, we explored to what extent the time per tap depended on the condition in the previous trial, the choice made on the previous trial (if there was a choice), and whether one had managed to hit the target on the previous trial. For this analysis of serial dependence we use the median time per tap, without correcting for the fraction of misses, because we are interested in how participants modify their behaviour on a trial-to-trial basis rather than in performance in terms of targets hit per time unit.

## Results and discussion

**Fig. 9:**
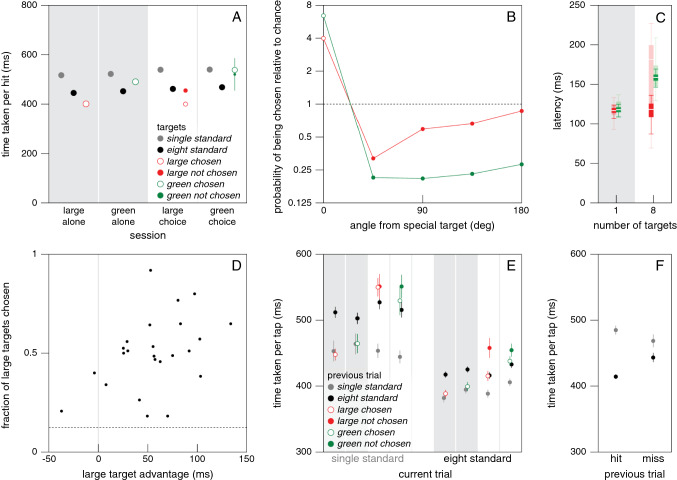
Results of Experiment 4. Error bars are standard errors across 24 participants. **A**. Time taken per hit for each of the three conditions (single standard target, eight standard targets, condition with a special target) of each of the four sessions. In the choice sessions (white background), the areas of the open and solid coloured disks indicate the relative fractions of trials in which the special target was chosen. **B**. The probability of tapping on a target as a function of its position with respect to the special target in the choice sessions. **C**. Saturated colours: the latency of corrections to the finger’s path in the same format as in Fig. 7. Faint colours: latencies for the same conditions in Experiment 2. **D**. The fraction of large targets that each participant chose in the condition with one large target and seven standard targets (large choice session) as a function of the expected advantage of choosing the large target. The dashed line indicates chance performance. **E**. Time taken per tap for the two conditions that were present in all four sessions. The disk colours in this panel indicate the condition in the previous trial. The condition in the current trial is shown on the horizontal axis. These are now median times taken per tap rather than per hit. The colours of the background correspond to the sessions as in panel A. **F**. Time taken per tap for the same two conditions, split by whether the previous trial was a hit or a miss

As was to be expected on the basis of Experiments 2 and 3, it took less time to hit one of eight targets than to hit a single identical target (black disks below grey disks in Fig. 9A). Importantly, for the *standard target conditions* the time per tap depended on whether there was a choice in the condition with the special target (*alone* versus *choice* sessions in Fig. 9A), but not on the kind of special target (*large* or *green)*. This was the case for both the *single standard target condition* (alone or choice: *F*_1,23_ = 6.05, *p* = 0.022; large or green: *F*_1,23_ = 0.13, *p* = 0.73; interaction: *F*_1,23_ = 0.052, *p* = 0.82) and the *eight standard target condition* (alone or choice: *F*_1,23_ = 13.93, *p* = 0.0011; large or green: *F*_1,23_ = 1.46, *p* = 0.24; interaction: *F*_1,23_ = 0, *p* = 0.99). Participants took significantly longer to hit standard targets in the choice sessions (Fig. 9A; the difference is 20 ms for the *single standard target condition* and 16 ms for the *eight standard target condition*). Before examining whether this small difference arises from adjusting one’s behaviour to the whole set of conditions within the session or to the previous trial, we confirm that the results are otherwise similar to those of Experiments 2 and 3.

As expected, it took less time to hit large targets or one of eight standard targets than to hit a single standard target (Fig. 9A). The observation that we made in Experiment 2 (Fig. 5C) that participants took less time per hit for targets that were associated with more points was also replicated: single green targets took less time than spatially identical single standard targets (*green alone session*). Again participants both moved faster and missed fewer targets, confirming that participants can do better if they know that it is more important (Manohar et al. 2015; Summerside et al. 2018). As anticipated, participants usually chose the green target (*green choice session*). They no longer only chose the large target at the expense of targets that were within 90 deg (*large choice session*), presumably because the large target was now larger than in the previous experiments. Nevertheless, neighbouring targets were still chosen less frequently (Fig. 9B).

The time taken to select the green target and adjust the finger’s path to this choice was similar to the time taken in Experiment 2 (green bars in Fig. 9C). It was again slightly longer than the time taken to adjust the path to the position of a single green target appearing. The additional time taken to adjust the movement might explain why it did not take less time to hit a chosen green target than a single standard target (*green choice session*), although it did take less time to hit a single green one (*green alone session*). One difference that we observed between the results of this experiment and those of Experiments 2 and 3 is that here the time taken to adjust the finger’s path towards the large target when there were eight targets was no different from the time taken when the large target was presented on its own (red bars in Fig. 9C). The correlation across participants between the expected advantage of choosing the large target and the actual frequency with which the large target was chosen was 0.42 (*t*_22_ = 2.19, *p* = 0.04; Fig. 9D), which is similar to the correlations obtained in Experiments 2 and 3, but here it is significant.

Having confirmed that performance is generally consistent with that of the previous experiments, we examined why the time taken per hit is slightly larger in the *choice sessions* than in the *alone sessions* (Fig. 9A) by determining how the time per tap depends on the condition, choice and outcome of the previous trial. The most evident influence of the previous trial is that it took less time to hit targets when there was a single target in the previous trial (all grey disks and red and green disks on grey backgrounds in Fig. 9E) than when there were eight targets in the previous trial (all black disks and red and green disks on white backgrounds; note that in Fig. 9E the disk colours indicate the condition during the *previous* trial). Considering the magnitude of this effect, the slightly larger time taken per hit in the *choice sessions* than in the *alone sessions* (Fig. 9A) is primarily the result of there being more previous trials with eight targets in the *choice sessions* than in the *alone sessions*.

If there was no choice on the previous trial, the kind of target did not matter (red and green disks on the grey backgrounds were not systematically higher or lower than the corresponding grey disks in Fig. 9E). Thus, movements take longer after a trial with multiple targets, irrespective of the kind of target. This might be because when there are many targets participants have to make a choice. If so, the actual choice on the previous trial might also matter. The time taken per hit was consistently longer after trials in which participants did not choose the special target (solid red and green disks higher than corresponding black disks). This might be because participants regarded not choosing the special target to be an error. For the *eight standard target condition* (for which we have the most data), it is clear that participants took longer to tap after having missed the previous target than after having hit it (black disks in Fig. 9F), as was to be expected after an error (Brenner and Smeets 2011). For some reason this was not the case for the *single standard target condition* (grey disks), but altogether the results suggest that participants slow down after what can be considered to be an error.

In accordance with the choice rather than target identity being important, having chosen the special target on the previous trial (open disks on white background in Fig. 9E) was equivalent to having chosen one of eight standard targets (black disks) when tapping on one of eight targets. However, when tapping on a single target, having chosen the special target on the previous trial resulted in times per tap that may be longer than after having chosen one of eight standard targets, though probably not as long as after not having chosen the special target (open disks between solid red and green disks and black disks). Thus, both the number of choices and ‘errors’ in the previous trial appear to influence performance. Slowing down after making a suboptimal choice (choosing one of the standard targets when one could have chosen the special target; solid red and green disks in Fig. 9E) probably contributes to the time taken to tap on targets being longer in the two conditions that were present in all four sessions when the third condition contained a choice between different kinds of targets. However, the main reason for taking longer in those sessions is that the time per hit is longer after trials with eight targets than after trials with only one, and there are more trials with eight targets in the choice sessions than in the alone sessions.

## General discussion

The goal of the present study was to determine whether selecting between simple movement options takes a substantial amount of additional time, and if so under what circumstances. Finding that the response latency hardly depends on the number of options that need to be considered confirms the assumption that multiple options are evaluated simultaneously. Moreover, it suggests that selecting the most suitable option on the basis of such evaluations takes a negligible amount of additional time (for a review of additional evidence see Gallivan et al. 2018). We were particularly interested to know whether this was only possible for information that normally guides movements. The results show that selecting an option on the basis of an arbitrary association of colour with points also takes almost no additional time, even without extensive training, although the choice may sometimes be slightly less efficient than when considering the target’s size. Thus, the ability to efficiently select one of many options is probably quite a general feature of motor control.

It generally took almost as long to adjust movements when there was no choice as when there was a choice. In Experiment 1, the difference in response latency between trials with and without a choice was negligible for a choice between 2 options that differed in position and size (Fig. 3C). In Experiments 2 and 3 (Figs. 7 and 8C), the response latency appeared to be longer when there were several options, but the judged latencies were very imprecise. In Experiment 4, the difference was negligible when there were eight options and the choice was based on size (shown in red in Fig. 9C), but it was about 50 ms when the choice was based on colour (shown in green in Fig. 9C). That the difference was not negligible for colour could be because colour arbitrarily determined the awarded points, whereas size directly determined the required precision of the movement endpoint. However, it is more likely that when there was a single coloured target participants adjusted their movement faster because they did so on the basis of the target’s position without considering its colour. Adjustments to changes in a target’s position take longer if only the target’s colour distinguishes it from the background (Brenner and Smeets 2003), presumably because colour-processing pathways are slower and have less direct connections with brain areas that guide our actions (Goodale and Milner 1992). In accordance with it taking longer to respond to colour (also see Appendix 3 and Veerman et al. 2008) but the options still being processed simultaneously, the latency with which the movements were adjusted towards the green target increased very gradually, if at all, with the number of options in Experiment 2 (data shown in green in Fig. 7).

It is not clear why the latency with which participants adjusted their movements towards the larger target appeared to be longer when there were more than 2 targets in Experiments 2 and 3. This might somehow be related to the lack of precision as a result of the lower number of trials per condition (and of participants for Experiment 3) in combination with the complicated method of determining the latency. Alternatively, the fact that there were more conditions in Experiments 2 and 3 might force participants to consider additional information. When a spherical object that they were reaching out for suddenly rotated, participants briefly adjusted their movements in accordance with the displacements of the grasping points on the object’s surface before reconsidering how to grasp the object (Voudouris et al. 2013). Presumably, reconsidering where to place their fingers on the object took more time than updating the positions of the original grasping points, even though participants were free to grasp the object in any way they liked. When the target of an ongoing movement was replaced by two new options it also took about 50 ms longer to adjust the movement towards one of them (usually the one that was nearer) than when the target was replaced by a single new option (Kurtzer et al. 2020). It is not evident that more information was required in that study than in the present one, but maybe the fact that there were nine possible target configurations made a difference. The fact that we show that choices can be made near-instantaneously under some circumstances, irrespective of the number of options, suggests that it might be more fruitful to search for differences in the information that is considered, and how long it takes to process such information, rather than interpreting latency differences as the time needed to pick one of the options. Such an approach might even explain why some information is not considered at all, such as the biomechanical costs of goal-directed arm movements (Michalski et al. 2020). It might take too long to process such information (although it might also simply not be considered relevant for the task).

Of course, interpreting a lack of differences in latency as showing that the choice itself is made very quickly is only meaningful if the choices are reasonable rather than arbitrary. We show that participants choose targets that are easier to reach due to their position, size or orientation. This is evident from the frequencies with which various targets were chosen, but also from the fact that the time taken per tap was shorter when there was more than one target, even when all the targets were identical. When the advantage arose from arbitrarily assigning more points to a target of a certain colour participants also often chose that target, but probably less often than they should have done to maximize their points. Moreover, individual participants’ choices appeared to depend on how they performed for the various targets involved, because there was a positive correlation between their choices and our estimates of the benefit of making certain choices (although it was only significant for Experiment 4).

The idea that it takes people a negligible amount of additional time to guide their movements when they have several options to select from, even when there is an arbitrary relationship between some aspect of the stimulus and the correct response, seems strange considering that there is a long history of finding and using differences in reaction time to evaluate how long it takes to make various distinctions (Donders 1969; Hyman 1953; Merkel 1885; Teichner and Krebs 1974). One reason why choices are much faster in this study is probably that they were made by moving the same finger to different positions, rather than by moving different fingers for different choices (Wright et al. 2019). Another potential reason is that our method emphasized speed, with selecting an incorrect target only implicitly being regarded as an error. Indeed, our participants made many ‘errors’ when colour indicated that a target was worth more points: they would have obtained more points if they had chosen the green target more often. In traditional choice reaction time studies, selecting the incorrect target is strongly discouraged, so participants may delay their choices by considering all options sequentially to reduce the number of errors.

For most choices in daily life, such as deciding whether one should stop or speed up when the traffic lights change colour, the relationship between the options and the rewards is not arbitrary, but it is complicated. It even depends on how quickly one can choose, because the merits of the options change as time progresses. We tried to design our task in a manner that makes it easy to evaluate whether choices are reasonable. We found that they are, but we also found some serial dependence (see Fig. 9E) that we do not immediately understand in terms of improving performance. Such serial dependence might arise from the mechanism by which movement speed is optimized (Brenner and Smeets 2011). It is unlikely to be the consequence of also considering future movements (Hoppe and Rothkopf 2019), because if so movements would presumably have been faster rather than slower after having many options. Having many options would presumably allow participants to place their hand at a position that will make the next movement easier. We conclude that people make reasonable, near-instantaneous choices irrespective of the features involved, although some features do take longer to respond to. Various instantaneous circumstances are considered when making the choice, but the choice is also influenced by the circumstances in the previous trial.

## Figures and Tables

**Fig. 10:**
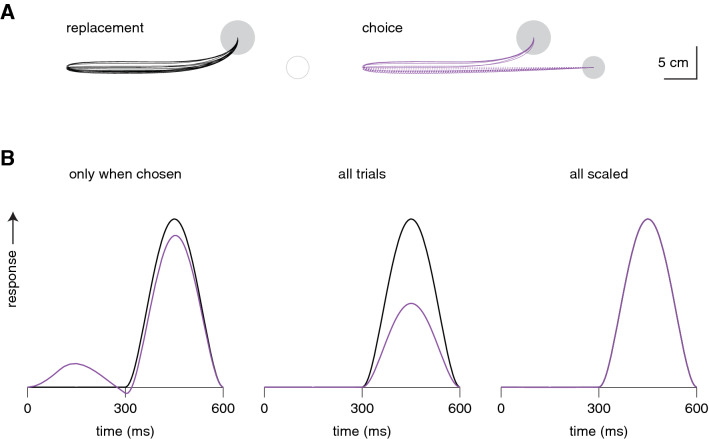
Simulations for the two main conditions of Experiment 1. **A**. Trajectories of twenty trials (black and purple curves) in which the movement started in a random direction, then (after 100 ms) moved towards an initial small target on the right (the small grey circle or disk), and was finally (after another 200 ms) diverted towards a new larger target (the large grey disk) on replacement trials and on half of the choice trials. In the choice condition, whether the movement was diverted towards the new target depended on the initial random movement. **B**. Average response (velocity orthogonal to the direction to the original target, towards the new target) for 1000 simulations in each of these conditions. For the choice condition (purple curve), if only trials in which the new target is chosen are averaged (only when chosen) there appears to be a response towards the new target before the simulated response starts (during the first 300 ms). Averaging all trials removes this selection artefact (all trials), but reduces the response amplitude. Scaling the response by the fraction of trials in which the new target was chosen yields the response that we simulated (all scaled). Such selection and scaling does not influence the response in the replacement condition, because all movements are diverted when the target is replaced

**Fig. 11:**
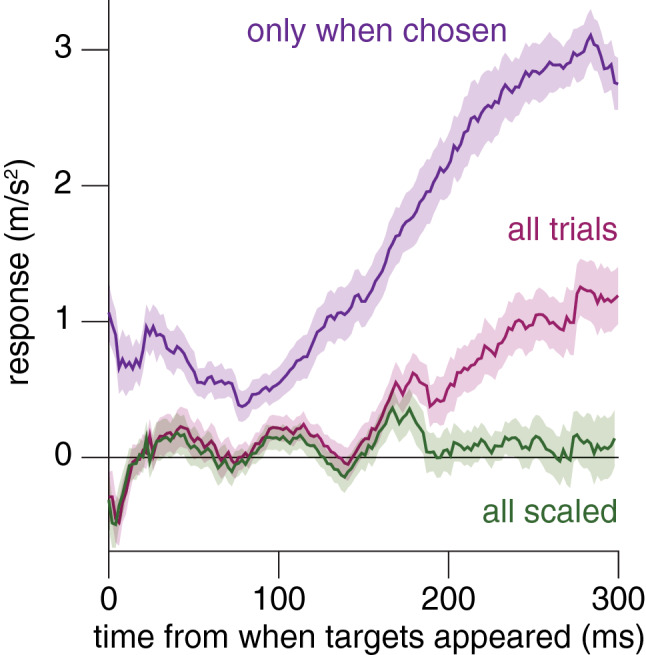
Illustrating why we cannot use a simpler method to calculate the response based on target identity. We took the data in the condition with 8 identical target disks in Experiment 2 and randomly selected one of the targets to be the special target. Obviously, analysing this data should not reveal a response, because there cannot be a response based on selectively moving to a certain kind of target since there is only one kind of target. If only trials in which the randomly selected target was chosen are considered, there is a clear response towards that target (purple curve). This is not surprising, because whether a target is chosen depends on how one is moving when the choice is made, and the movement usually has to be adjusted to reach the chosen target, irrespective of why that target was chosen. The response is largely removed by considering all trials, but it is not removed altogether (red curve). There is still some response because as the movements curve towards one of the targets, extrapolating the instantaneous movement does not split the targets into two equal groups. Generally, more targets are at the side towards which the movement curves. The residual response can be removed by scaling individual responses by the fraction of targets for which the measured response would be in the wrong direction (green curve)

**Fig. 12:**
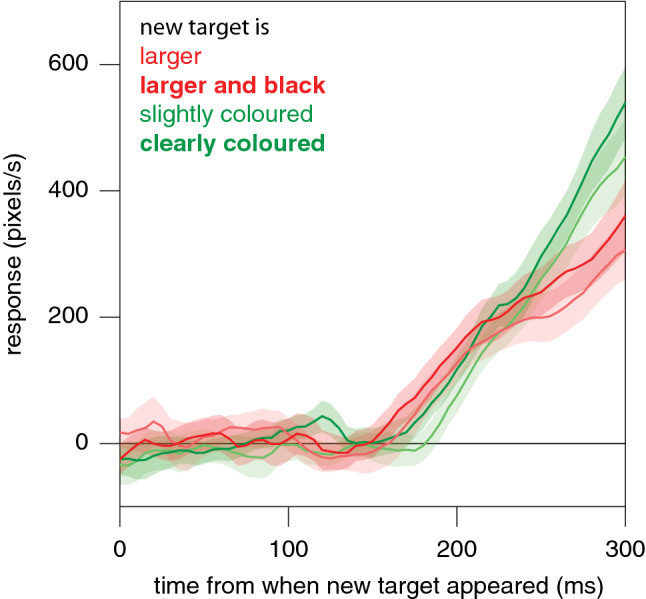
Average responses (with standard errors across participants) for the four kinds of new targets

## Data Availability

The datasets generated and analysed during the current study are available from the corresponding author on reasonable request.

## Appendix

### Appendix 1: Averaging responses in Experiment 1

To illustrate the artefacts that would emerge if only responses on trials in which participants chose the new target were averaged, and to explain why averaging all trials and scaling the outcome is a good way to calculate the response, we simulated movements for the two main conditions of Experiment 1 and analysed them in the same way as we did the experimental data. We simulated minimum-jerk movements that last 600 ms. Participants started moving towards a position 5 cm away from the starting point in a random direction. After 100 ms, the movement was diverted towards the original target (Flash and Henis 1991), 35.2 cm from the starting point. The only source of variability in the simulated movement trajectories is the random direction of motion during the first 100 ms, before the movement could be directed towards the original target (Fig. 10A). After another 200 ms, the movement could be diverted towards a new target that was 10 deg counterclockwise at a distance of 26.4 cm from the starting point. In simulations of the replacement condition, all movements were diverted to this new target. In simulations of the choice condition, half the movements were diverted: movements that initially moved more than 5 deg (and less than 185 deg) counterclockwise with respect to the direction towards the original target.

As the simulated response was the same for the diverted movements of both conditions, a correct analysis should give the same response for both. In the choice condition, the choice between targets depends on the random initial movement, so the average of the trials in which the new target was chosen differs from the start. This gives rise to an artefact (*only when chosen* in Fig. 10B): there appears to be a response before the new target appears. Moreover, the amplitude of the response at the moment at which it is expected is reduced. The artefact can be removed by averaging *all trials*, but this obviously results in the response amplitude being smaller, because trials with no simulated response are included in the average. We can correct for this by dividing the average response of all trials by the fraction of trials in which the new target was chosen (*all scaled* in Fig. 10B). Doing so gives the same response for both conditions.

### Appendix 2: Averaging responses in Experiments 2-4

One of our goals in Experiments 2–4 was to determine the latency of responses based on target identity. To do so, we had to isolate responses based on target identity from simply curving towards the nearest target. This cannot be achieved by selecting trials in which the movement ends on the special target, because the finger’s movement is always adjusted towards the target on which the movement ends, and acceleration that curves the path towards the target must be considered positive (irrespective of the side from which the finger approaches). To illustrate the problem, we randomly selected one of the eight targets of all trials in the condition with 8 standard (circular) targets of Experiment 2, and treated it as if it were a special target. Obviously, there can be no response based on target identity for a randomly selected target. Nevertheless, averaging the acceleration orthogonal to the direction of motion for the movements that ended on the randomly selected target gives rise to a clear response (purple curve in Fig. 11B). Some response is even visible as soon as the targets appear, which must be because how the finger is moving at that moment influences the choice (a selection artefact). Thus, selecting trials in which the special target is chosen cannot isolate responses based on target identity.

Considering all the trials, irrespective of which target was chosen, but analysing the response with respect to the (randomly) selected target, removes the selection artefact and partially removes the effect of curving towards the nearest target irrespective of its identity (red curve in Fig. 11). It does not completely remove the effect of curving towards the nearest target, because although acceleration orthogonal to the instantaneous direction of motion makes the movement curve towards some targets and away from others, extrapolating the instantaneous movement does not always split the targets into two equal groups. Due to the target layout, there are usually more targets in the direction in which the trajectory curves than in the opposite direction, so more positive than negative responses overall, even if there is no specific tendency to choose the (randomly) selected target. To compensate for this bias, we scale the orthogonal acceleration by the fraction of targets for which that acceleration would curve the movement away from the target. Doing so does a reasonable job of removing the bias (green curve in Fig. 11). Analysing the data in this manner, we see no response based on target identity when all targets are identical so that there can be no response based on target identity. In conditions in which participants often direct their movements towards a particular target (the special targets of Experiments 2–4), responses do not cancel out when averaging scaled accelerations with respect to the position of the selected target (Fig. 6).

### Appendix 3: Supplementary experiment

To examine whether people can just as quickly switch to a new target when the new target is only advantageous because it is associated with more points (as indicated by its colour) we conducted a supplementary experiment that was similar to Experiment 1. In this supplementary experiment participants moved a cursor to targets on a screen using a computer mouse. We compared responses to larger new targets (as in Experiment 1) to ones to differently coloured new targets that were worth more points. We also examined whether making the new targets more conspicuous, as might automatically be the case when they are larger, makes a difference.

Twenty participants each performed 5 sessions, each lasting 90 s, of which the first was considered practice. Rather than using a fixed delay at which new targets appeared, we used a staircase to determine the delay for which participants switched on 50% of the trials. Each kind of new target had a separate staircase. The delay for each kind of new target increased by 20 ms if the target was chosen, and decreased by 20 ms if it was presented but not chosen. The practice session ensured that the staircases in the sessions that were analysed started close to the 50% switch threshold, because although the delay started at a fixed value of 300 ms for each kind of target for every participant, it continued from the last attained value on the 4 subsequent sessions for the same target and participant. There was a short break between sessions.

Within each session the participant had to move a cursor to a target by moving a computer mouse. Once the cursor was within a target for 50 ms (to force participants to stop within the target) a new original target appeared at a fixed distance (but random direction) from it. On each trial there was an independent 20% probability of a second target appearing. Such a target was always at the same distance from the previously attained target as the original target, but in a direction that was either 15 deg clockwise or counterclockwise (chosen at random) with respect to the original target. The original target was grey on a white background. The new target could be grey but have twice the diameter (24% rather than 12% of the distance to the target), it could have twice the diameter and also be black, making it more conspicuous, it could be the same size as the original target but bright red, or it could be the same size and only slightly reddish. Participants were told that red or reddish targets were worth double points. Their task was to obtain as many points as possible within each session.

Movements of the computer mouse were polled at 1000 Hz. To evaluate the latency of the response, the velocity was determined using a second-order Savitzky–Golay filter with a window size of 20 ms. For trials in which a new target appeared, the component of the velocity that was orthogonal to a straight line from the target of the previous movement to the original target of the current movement was isolated. This velocity was inverted if the new target appeared clockwise with respect to the movement, so that a response in the direction of the new target was always positive. Since the cursor was only redirected to the new target on half the trials, the resulting response is about half the magnitude of responses on trials in which the cursor was redirected.

The delay at which participants switched on 50% of the trials was about 250 ms for the large targets and about 300 ms for the reddish ones, with possibly a slightly higher value when the luminance or colour contrast was high than when it was low. That the delays at which participants switched to the other target were different for different targets confirms that participants did not simply switch whenever a new target appeared. More importantly, the latencies of the responses to the four kinds of new targets were similar. The latencies are actually shorter than one would estimate from Fig. 12, because there were delays in our system that we did not consider. Since such delays do not depend on the size or colour of the target the relative latency is reliable. That the latency is slightly longer for responding to colour than to size is consistent with previous findings, as is the slightly shorter latency for more conspicuous targets (Veerman et al., 2008). Thus, there is no indication that it takes fundamentally longer to respond to an arbitrary association between colour and value (in points) than it does to respond to the target’s size (that is relevant for the movement itself) when making a choice.
